# Clinical efficacy of guided tissue regeneration combined with orthodontic treatment on periodontitis

**DOI:** 10.3389/fbioe.2026.1803582

**Published:** 2026-04-21

**Authors:** Fengjuan Wang, Feng Yang, Chun Yao, Yanxiao Du

**Affiliations:** 1 Qingdao Central Hospital, University of Health and Rehabilitation Sciences (Qingdao Central Hospital), Qingdao, Shandong, China; 2 Department of Stomatology, the Affiliated Hospital of Xuzhou Medical University, Xuzhou, Jiangsu, China; 3 School of Stomatology, Xuzhou Medical University, Xuzhou, Jiangsu, China; 4 Department of Stomatology, Zhenjiang First People’s Hospital, People’s Hospital Affiliated to Jiangsu University, Zhenjiang, Jiangsu, China; 5 The Second Affiliated Hospital of Guangdong Medical University, Zhanjiang, Guangzhou, China; 6 Changchun Institute of Applied Chemistry Chinese Academy of Sciences, Changchun, Jilin, China; 7 WEIGO Holding Company Limited, Weihai, Shandong, China

**Keywords:** clinical efficacy, guided tissue regeneration, NLRP3, orthodontic treatment, periodontitis

## Abstract

**Background:**

Periodontitis is a common chronic inflammatory disorder caused by bacterial plaque. Our study aims to evaluate the clinical efficacy of guided tissue regeneration with orthodontic treatment (GTRO) in patients with periodontitis.

**Methods:**

Propensity score matching was performed based on gender, age, periodontitis duration, dental malformation, sulcus bleeding index, and gingival index. Clinical efficacy, gingival index (GI), sulcus bleeding index (SBI), probing depth (PD), clinical attachment loss (CAL), and depth of bone defect were assessed 6 months post-treatment. The NLR family pyrin domain containing 3 (NLRP3), IL-1β, and IL-18 levels in gingival crevicular fluid (GCF) were determined using ELISA.

**Results:**

A total of 131 patients were included in the final analysis, including 64 in the GTR group and 67 in the GTRO group. The GTRO group demonstrated better outcomes, including higher recovery and total efficacy rates. Post-treatment, the GTRO group exhibited greater improvements in GI and SBI, PD, CAL, and depth of bone defect than the GTR group. Levels of NLRP3, IL-1β, and IL-18 in GCF significantly decreased in both groups, with more pronounced reductions observed in the GTRO group.

**Conclusion:**

GTRO improved clinical and anti-inflammatory outcomes and was associated with better periodontal health and bone defect repair than GTR alone at 6 months.

## Introduction

Periodontitis is a prevalent chronic inflammatory disorder of the oral cavity, with a global prevalence estimated between 20% and 50% (Di Stefano et al., 2022), and an incidence that increases with advancing age. Chronic periodontitis poses a major burden on global oral health and is primarily caused by bacterial infections within dental plaque (Ganimusa et al., 2024; Morandini and Ramos-Junior, 2024), which leads to the degradation of periodontal tissues, including the gingiva, periodontal ligament, and alveolar bone (Feng et al., 2024).

The pathogenesis of periodontitis is driven by an imbalance between pathogenic bacteria and the host’s immune response (Saliem et al., 2022), a process that is potentiated by the accumulation of dental biofilm. This biofilm serves as a reservoir for pathogens, thereby triggering and sustaining inflammation (Slots, 2017). The immune system subsequently releases both pro-inflammatory and anti-inflammatory cytokines in response to the infection (Ebersole et al., 2017). Interleukin (IL)-1β is a pro-inflammatory cytokine that contributes to tissue degradation in periodontitis (Pan et al., 2019). Its levels in gingival crevicular fluid (GCF) rise rapidly with the onset of inflammation, originating from its release by various cells in response to periodontal pathogens (Salah and Abdulbaqi, 2023). Patients with periodontitis show elevated IL-1β concentrations in their serum, saliva, and gingival crevicular fluid (GCF), with the highest levels often observed in GCF. IL-18 is involved in several inflammatory diseases, including Crohn’s disease, celiac disease, type 2 diabetes, and periodontitis (Ajdani et al., 2022; Li et al., 2023). Higher serum levels of IL-18 have been found in patients with both inflammatory bowel disease and periodontitis (Techatanawat et al., 2020). The NLR family pyrin domain containing 3 (NLRP3) inflammasome is a critical mediator of the innate immune response, activated by various stimuli like infections and tissue damage (Lamkanfi et al., 2009). It triggers the release of pro-inflammatory cytokines, such as IL-1β and IL-18, which are involved in inflammation (Yang et al., 2015). In periodontitis, NLRP3 activation in response to bacterial pathogens contributes to chronic inflammation and tissue destruction (Olsen and Yilmaz, 2016). Dysregulated NLRP3 signaling may exacerbate periodontal inflammation, suggesting that it represents a promising therapeutic target for periodontitis.

For periodontitis, basic periodontal therapy and guided tissue regeneration are commonly used to remove plaque and induce periodontal tissue regeneration (Kumar and Madurantakam, 2017). Given the complex and delicate structure of periodontal tissues, improper therapeutic approaches may result in adverse outcomes, including tooth mobility, tooth loss, and complications such as malalignment or displacement of teeth. Moreover, guided tissue regeneration (GTR) poses a risk of bacterial contamination, potentially disrupting the correction of dental morphology and alignment, and ultimately hindering the functional recovery of periodontal tissues (Isola et al., 2018). Orthodontic treatment is effective in promoting tooth alignment and the establishment of functional occlusion. It also plays a positive role in enhancing wound healing and facilitating the regeneration of periodontal tissues. Recent studies have shown that orthodontic treatment can significantly increase alveolar bone height and reduce periodontal pocket depth in patients with periodontitis after periodontal inflammation has been controlled (Ishihara et al., 2015). In this study, we retrospectively evaluated the clinical outcomes of guided tissue regeneration combined with orthodontic treatment (GTRO) in patients with periodontitis, with a focus on improvements in periodontal health and the alteration of NLRP3, IL-1β, and IL-18 levels in GCF. The goal is to enhance clinical efficacy while also improving dental aesthetics and masticatory function.

## Materials and methods

### Participants

A retrospective study was performed to compare the outcomes of GTR and GTRO. Data were retrospectively collected from patients who underwent GTR or GTRO at our department over the past 3 years. Propensity score matching was used to reduce baseline differences between the two groups. After matching, 64 patients were included in the GTR group and 67 in the GTRO group. The study was approved by the Second Affiliated Hospital of Guangdong Medical University. Written informed consent was waived as this was a retrospective analysis.

### Inclusion criteria

Patients were eligible for inclusion if they met the following criteria: age ≥18 years; a clinical diagnosis of periodontitis with probing depths ≥5 mm following initial therapy; tooth mobility ≤ grade II; and provided written informed consent.

### Exclusion criteria

Patients were excluded if they met any of the following criteria: other oral diseases such as pulpitis; tooth mobility or malformation due to traumatic injury; significant dysfunction of vital organs; history of malignancy or infectious diseases; or being pregnant or breastfeeding.

### Treatment

Both groups of patients initially underwent basic periodontal therapy, including supragingival scaling, subgingival curettage, and root planing, along with oral health education to ensure proper toothbrushing techniques.

On this basis, patients in the GTR group subsequently underwent GTR as follows. The surgical procedure was indicated for patients with probing depths exceeding 5 mm and radiographic evidence of angular bone resorption. In cases of severe alveolar bone defect, an artificial bone graft was placed at the alveolar crest. A mucoperiosteal flap was then repositioned and sutured, with complete removal of the compromised gingival tissue.

For the GTRO group, orthodontic treatment was initiated 3 months following GTR. The procedure included the application of buccal tubes to the molars for anchorage, and the bonding of rectangular brackets to the anterior teeth to enhance support. An orthodontic appliance was subsequently inserted, and alignment was achieved through the use of nickel-titanium archwires. A light-force protocol was used for patients with periodontitis, with an initial force of 50–100 g, which was later adjusted to 100–150 g according to tooth movement and periodontal status. A standardized archwire sequence was applied, consisting of 0.014-inch, 0.016-inch, and 0.018-inch round nickel-titanium wires, followed by a 0.019 × 0.025-inch rectangular nickel-titanium wire for alignment and space adjustment. Force was maintained for 3–4 weeks after each activation. The duration of orthodontic treatment before the 6-month efficacy evaluation was 3 months. Post-treatment care included tooth cleaning every 2–3 days, weekly periodontal assessments, and meticulous maintenance of the orthodontic appliance. Follow-up appointments were scheduled every 3 months for appliance adjustments.

### Efficacy evaluation

Clinical efficacy was evaluated 6 months after the initial treatment, according to the following criteria. Recovery: Complete resolution of clinical symptoms, including periodontal bleeding, swelling, and pain. The gingival tissue appeared pink and resilient, with no bleeding on probing, and radiographs showed bone hardening with no alveolar bone resorption. Effectual: Significant improvement in periodontal bleeding, swelling, and pain. Gingival tissue showed noticeable changes, with slight bleeding on probing, and radiographs indicated a marked reduction in alveolar bone resorption. Effective: Mild improvement in periodontal bleeding, swelling, and pain. Gingival tissue appeared red, swollen, and lax, with bleeding on probing. Radiographs showed no significant change in alveolar bone resorption. Ineffective: No change or worsening of clinical symptoms and gingival condition, with radiographs showing no change or further progression of alveolar bone resorption.

The total efficacy rates were calculated as follows: Total efficacy rates = (Recovery + Effectual + Effective)/Total number of cases × 100%

### Gingival index (GI) assessment

The GI was measured before treatment and at 6 months post-treatment to evaluate changes in gingival texture, color, and bleeding tendency. A four-point scale was used, ranging from 0 to 3, where 0, 1, 2, and three correspond to healthy gingiva, mild inflammation, moderate inflammation, and severe inflammation, respectively.

### Sulcus bleeding index (SBI)

The SBI was assessed using the following scoring system.

0 = Healthy gingiva, no inflammation or bleeding;
1 = Gingival color shows inflammatory changes, no bleeding on probing;
2 = Spot bleeding upon probing;
3 = Bleeding extending along the gingival margin after probing;
4 = Bleeding fills the sulcus and overflows;
5 = Spontaneous bleeding.

### Probing depth (PD) index

The PD index was defined as the distance from the gingival margin to the base of the periodontal pocket or gingival sulcus, measured during probing.

### Clinical attachment loss (CAL) index

The CAL index was defined as the distance from the cementoenamel junction to the base of the periodontal pocket.

### Depth of bone defect

Depth of bone defect was defined as the distance from the crest of the alveolar ridge to the deepest point of the bone defect.

### GCF collection and analysis

GCF samples were obtained before treatment and at 6 months post-treatment. The collection procedure was as follows: Patients were first instructed to rinse their mouths with water to remove any residual food debris. After thoroughly drying the tooth surfaces and gingival tissues, a filter paper strip was inserted into the distal buccal gingival sulcus of the affected tooth. The insertion was halted upon encountering resistance, and the strip was left in place for 30 s. The strip was then carefully transferred to a centrifuge tube. A second sample was collected from the same site after 10 min, and both samples were subsequently centrifuged. The GCF was stored at −80 °C until analysis.

The concentrations of NLRP3 (EH4202, FineTest, Wuhan, China), IL-18 (EH0011, FineTest), and IL-1β (EH0185, FineTest) in GCF were determined using enzyme-linked immunosorbent assay (ELISA) kits obtained from commercial company. The ELISA procedures were performed according to the manufacturer’s instructions. The analytical performance of the ELISA kits was as follows: for NLRP3, the assay range was 0.781–50 ng/mL, the sensitivity was 0.469 ng/mL, and both the intra- and inter-assay coefficients of variation (CVs) were <6%; for IL-1β, the assay range was 3.906–250 pg/mL, the sensitivity was 2.344 pg/mL, and both the intra- and inter-assay CVs were <5%; for IL-18, the assay range was 15.625–1000 pg/mL, the sensitivity was 9.375 pg/mL, and both the intra- and inter-assay CVs were <6%. GCF samples were analyzed without normalization to total protein.

### Statistical analysis

Data were presented as mean ± SD. Propensity score matching (PSM) was performed using R Studio (Version 1.4.1106) to reduce baseline differences between the two groups. Treatment group was used as the binary dependent variable (GTR group = 0, GTRO group = 1), and gender, age, duration of periodontitis, type of dental malformation, sulcus bleeding index, and gingival index were included as independent variables in a logistic regression model to estimate propensity scores. A 1:1 nearest-neighbor matching method without replacement was applied with a caliper width of 0.05. Covariate balance after matching was assessed using standardized mean differences (SMDs), with an SMD <0.1 considered indicative of acceptable balance.

A *post hoc* power analysis was conducted using G*Power software (version 3.1). The total efficacy rates were predefined as the primary clinical outcome. Based on the observed difference between groups, a total sample size of 131 patients (64 vs. 67) and a two-sided α level of 0.05 yielded a statistical power (1-β) of 0.82, indicating adequate power to detect between-group differences.

Effect sizes for categorical outcomes were expressed as risk ratios (RRs) with corresponding 95% confidence intervals (CIs), including total efficacy rates, improvement in sulcus bleeding index (defined as SBI grades 0–1), and favorable gingival status (defined as GI grades 0–1).

After matching, between-group comparisons were performed using two-way repeated measures ANOVA, Mann-Whitney test, Chi-square test or Fisher’s exact, as appropriate. A two-sided P < 0.05 was considered statistically significant.

## Results

### Inclusion procedure of periodontitis patients received the treatment of GTR or GTRO

**FIGURE 1:**
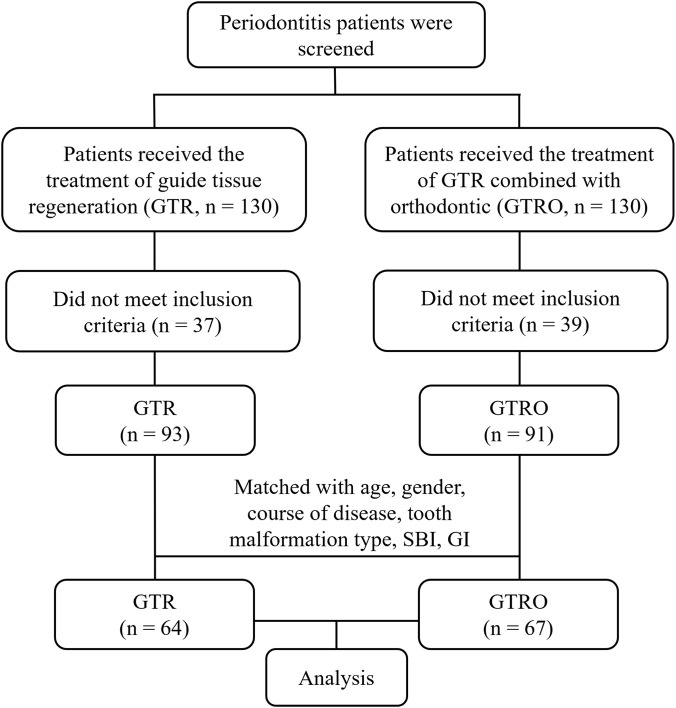
Inclusion procedure of periodontitis patients received GTR or GTRO.

A total of 260 periodontitis patients were screened and divided into two groups according to the treatment received. One group consisted of 130 patients who underwent GTR, while the other included 130 patients who received GTRO. After screening according to the inclusion criteria, 37 patients in the GTR group and 39 patients in the GTRO group were excluded because they did not meet the eligibility criteria, leaving 93 and 91 patients in the GTR and GTRO groups, respectively. To minimize potential confounding, patients in the two groups were matched based on age, gender, disease duration, type of dental malformation, SBI, and GI. After matching, 64 patients remained in the GTR group, and 67 patients remained in the GTRO group. These matched groups were then included in the final analysis (Figure 1).

### Demographic and clinical characteristics of periodontitis patients who received the treatment of GTR or GTRO

The GTR group, comprising 64 patients, and the GTRO group, comprising 67 patients, were well-matched in baseline characteristics. There were no significant differences in age or gender distribution (Table 1). The mean disease duration was similar between groups. Types of tooth malformation were comparable, with the most common being lingual tipping deep overbite (Table 1). Both groups showed similar distributions in tooth mobility grades (I and II), SBI, and GI scores (Table 1). Additionally, SMDs have been calculated to assess post-matching covariate balance, showing all SMDs less than 0.11. These results indicate that the GTR and GTRO groups were well-matched in terms of demographic and clinical characteristics, allowing for a balanced comparison of treatment outcomes.

**TABLE 1:** Demographic and clinical characteristics of periodontitis patients received GTR or GTRO.

Characteristics	Study group		p	SMD
	GTR (n = 64)	GTRO (n = 67)		
Age (years)	36.85 ± 6.36	37.12 ± 6.54	0.732	−0.04
Gender				
Male	35 (54.7%)	33 (49.3%)	0.601	0.11
Female	29 (45.3%)	34 (50.7%)		
Course of disease (months)	17.63 ± 3.17	17.29 ± 3.44	0.805	0.10
Type of tooth malformation				
Lingual tipping deep overbite	25 (39.1%)	24 (35.8%)	0.968	0.02
Maxillary protrusion	16 (25%)	19 (28.4%)		
Anterior crossbite	13 (20.3%)	13 (19.4%)		
Others	10 (15.6%)	11 (16.4%)		
Looseness				
I	31 (48.4%)	30 (44.8%)	0.728	0.06
II	33 (51.6%)	37 (55.2%)		
Sulcus bleeding index				
2	7 (10.9%)	6 (8.9%)	0.869	0.03
3	27 (42.2%)	25 (37.3%)		
4	25 (39.1%)	29 (43.3%)		
5	5 (7.8%)	7 (10.5%)		
Gingival index				
1	5 (7.8%)	7 (10.5%)	0.683	0.07
2	40 (62.5%)	37 (55.2%)		
3	19 (29.7%)	23 (34.3%)		

Values were expressed as n (percentage, %) or mean ± SD. p values were derived from Mann-Whitney test. Chi-square test or Fisher’s exact test was used for assessing distribution of observations or phenomena between different groups. SMD: standardized mean difference.

### Comparison of clinical efficacy between the two groups after treatment

The GTRO group exhibited a significantly higher recovery rate compared to the GTR group (29.8% vs. 18.7%, p = 0.016) as shown in Table 2. Effect size analysis demonstrated that the total efficacy rates was significantly higher in the GTRO group (RR = 1.18, 95% CI: 1.05–1.33), indicating a clinically meaningful advantage of combined periodontal–orthodontic therapy. In terms of specific efficacy classifications, a higher proportion of patients in the GTRO group achieved “Effectual” outcomes (41.8% vs. 26.6%), while the GTR group had a larger proportion of “Ineffective” cases (21.9% vs. 7.5%) as shown in Table 2. These findings suggest that GTRO presents superior clinical outcomes compared to GTR alone.

**TABLE 2:** Comparison of clinical efficacy between the two groups after the treatment.

Clinical efficacy	Study group		p
	GTR (n = 64)	GTRO (n = 67)	
Recovery	12 (18.7%)	20 (29.8%)	0.016
Effectual	17 (26.6%)	28 (41.8%)	
Effective	21 (32.8%)	14 (20.9%)	
Ineffective	14 (21.9%)	5 (7.5%)	
Total efficacy	50 (78.1%)	62 (92.5%)	0.025

Values were expressed as n (percentage, %). p value was derived from Chi-square test or Fisher’s exact.

### Comparison of SBI between the two groups after treatment

The GTRO group demonstrated a significantly higher proportion of patients in index 0, indicating the most favorable outcome, compared to the GTR group (29.8% vs. 15.6%, p = 0.045) as shown in Table 3. When improvement was defined as SBI grades 0–1, the GTRO group showed a significantly higher improvement rate than the GTR group (RR = 1.64, 95% CI: 1.25–2.15). Additionally, a greater percentage of patients in the GTRO group fell within index 1 (38.8% vs. 28.1%), while the GTR group had a higher distribution across indexes 2, 3, and 4, reflecting less favorable outcomes. These findings suggest that patients undergoing GTRO showed greater improvement in SBI compared with GTR alone.

**TABLE 3:** Comparison of sulcus bleeding index between the two groups after the treatment.

Sulcus bleeding index	Study group		p
	GTR (n = 64)	GTRO (n = 67)	
0	10 (15.6%)	20 (29.8%)	0.045
1	18 (28.1%)	26 (38.8%)	
2	16 (25%)	12 (17.9%)	
3	11 (17.2%)	6 (9.0%)	
4	9 (14.1%)	3 (4.5%)	

Values were expressed as n (percentage, %). p value was derived from Chi-square test.

### Comparison of GI between the two groups after treatment

Table 4 presents the comparison of GI scores between the GTR and GTRO groups following treatment. The GTRO group demonstrated a significantly greater proportion of patients achieving a GI of 0, indicative of optimal gingival health, compared to the GTR group (35.8% vs. 18.8%, p = 0.007, Table 4). When favorable gingival status was defined as GI grades 0–1, the GTRO group exhibited a significantly higher rate of improvement than the GTR group (RR = 1.61, 95% CI: 1.27–2.04). Additionally, the GTRO group had a higher percentage of patients in index 1 (43.3% vs. 32.8%, Table 4). In contrast, the GTR group had a greater proportion of patients with higher gingival index scores, with 28.1% in index 2% and 20.3% in index 3, as opposed to 14.9% and 6.0%, respectively, in the GTRO group. These findings suggest that patients receiving GTRO showed greater improvement in GI compared with GTR alone.

**TABLE 4:** Comparison of gingival index between the two groups after the treatment.

Gingival index	Study group		p
	GTR (n = 64)	GTRO (n = 67)	
0	12 (18.8%)	24 (35.8%)	0.007
1	21 (32.8%)	29 (43.3%)	
2	18 (28.1%)	10 (14.9%)	
3	13 (20.3%)	4 (6.0%)	

Values were expressed as n (percentage, %). p value was derived from Chi-square test.

### Comparisons of PD, CAL and depth of bone defect

**FIGURE 2:**
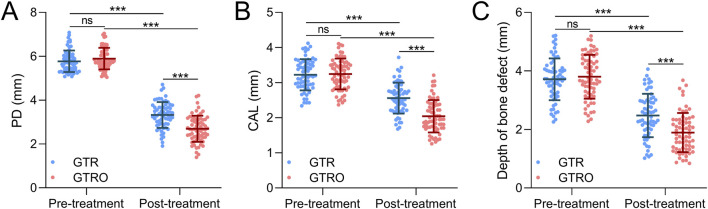
Comparisons of probing depth (PD, **(A)**, clinical attachment loss (CAL, **(B)**, and depth of bone defect **(C)** in periodontitis patients received the treatment of GTR (n = 64) or GTRO (n = 67) before and 6 months after the treatment. Values were expressed as mean ± SD. ***p < 0.001, and ns means no significance. Two-way repeated measures ANOVA analysis.

Both the GTR and GTRO groups showed significant reductions in PD after treatment compared to baseline (Figure 2A). In the GTR group, PD decreased from 5.770 ± 0.491 mm to 3.320 ± 0.589 mm, whereas in the GTRO group PD decreased from 5.891 ± 0.491 mm to 2.691 ± 0.597 mm. Similarly, CAL improved significantly in both groups post-treatment (Figure 2B). In the GTR group, CAL decreased from 3.224 ± 0.443 mm to 2.561 ± 0.440 mm, while in the GTRO group CAL decreased from 3.247 ± 0.440 mm to 2.042 ± 0.462 mm. In terms of depth of bone defect, both groups exhibited significant reductions after treatment (Figure 2C). In the GTR group, depth of bone defect decreased from 3.715 ± 0.714 mm to 2.476 ± 0.741 mm, whereas in the GTRO group it decreased from 3.801 ± 0.749 mm to 1.894 ± 0.667 mm. Overall, both treatment improved periodontal status, but GTRO achieved better outcomes.

### Comparisons of NLRP3, IL-1β and IL-18

**FIGURE 3:**
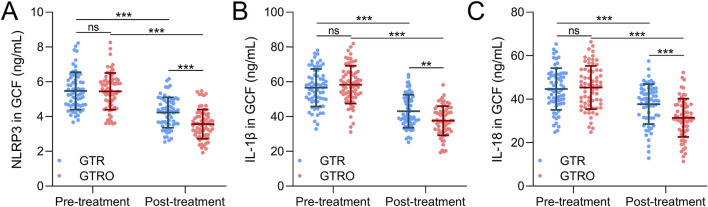
Comparisons of the concentrations of NLRP3 **(A)**, IL-1β **(B)**, and IL-18 **(C)** in GCF from periodontitis patients who received the treatment of GTR (n = 64) or GTRO (n = 67) before and 6 months after the treatment. Values were expressed as mean ± SD. ***p < 0.001, and ns means no significance. Two-way repeated measures ANOVA analysis.

Both treatment groups exhibited significant reductions in NLRP3 levels post-treatment compared to baseline (Figure 3A). In the GTR group, NLRP3 decreased from 5.475 ± 1.074 ng/mL before treatment to 4.229 ± 0.873 ng/mL after treatment. In the GTRO group, the level decreased from 5.449 ± 1.056 ng/mL to 3.567 ± 0.844 ng/mL. Similarly, IL-1β levels decreased significantly in both groups following treatment (Figure 3B). IL-1β levels declined from 56.571 ± 10.825 ng/mL to 43.078 ± 9.488 ng/mL in the GTR group, whereas they decreased from 58.317 ± 10.890 ng/mL to 37.600 ± 8.450 ng/mL in the GTRO group. A comparable trend was observed for IL-18 levels, with both groups showing substantial reductions post-treatment (Figure 3C). In the GTR group, IL-18 decreased from 44.654 ± 9.630 ng/mL before treatment to 37.702 ± 9.181 ng/mL after treatment. In the GTRO group, the level declined from 45.335 ± 9.952 ng/mL to 31.365 ± 8.807 ng/mL. Together, these findings indicate that NLRP3 inflammasome-related inflammatory markers were reduced in both groups after treatment, with greater reductions observed in the GTRO group compared with GTR alone.

## Discussion

This study evaluated the clinical efficacy of GTRO in periodontitis patients. The findings suggest that GTRO improved clinical outcomes, reduced inflammatory responses, and was associated with better short-term periodontal repair-related outcomes than GTR alone. These results demonstrate the synergistic benefits of GTRO, particularly in improving periodontal health, reducing inflammation, and enhancing tissue repair. GTR is typically performed prior to orthodontic tooth movement. Studies have demonstrated that whether orthodontic movement occurred in the early phase (less than 8 weeks) or late phase (more than 24 weeks) after GTR, both the clinical probing depth and attachment level of the affected teeth showed improvement (Tu et al., 2022). Early orthodontic movement post-GTR, conducted within immature bone tissue, has been found to stimulate periodontal tissue reconstruction, enhancing the efficiency of periodontal regeneration (Tu et al., 2022). Furthermore, when orthodontic tooth movement is directed toward periodontal bone defect areas, significant improvement in periodontal conditions is observed. Orthodontic tooth movement can facilitate GTR, which may also be performed after orthodontic treatment as needed (Passanezi et al., 2007).

In this study, the GTRO group demonstrated significantly higher recovery and efficacy rates compared to the GTR group. Specifically, a greater proportion of patients in the GTRO group achieved “effectual” outcomes, while the GTR group had a higher proportion of “Ineffective” cases. These findings are consistent with previous studies, which suggest that orthodontic treatment can enhance the overall outcome of periodontal regeneration (Nangia et al., 2024). By improving tooth alignment, orthodontic therapy helps stabilize the occlusion, reducing functional trauma on periodontal tissues and promoting more effective healing. Orthodontic treatment, combined with GTR, may also enhance the mechanical environment of the periodontium. Improved tooth alignment and occlusion can reduce occlusal forces, which are known to cause tissue damage in cases of malocclusion (Zhou et al., 2024). This stabilization effect contributes to better tissue regeneration, suggesting that GTRO can result in more favorable long-term periodontal outcomes compared to GTR alone.

Moreover, the study found that patients in the GTRO group exhibited significantly better outcomes in terms of SBI and GI, which are indicators of gingival health. The greater proportion of patients achieving optimal SBI and GI scores in the GTRO group suggests that orthodontic therapy may play a role in improving gingival health by alleviating local inflammation and promoting a more favorable periodontal tissue response. This could be due to the reduction in occlusal trauma and improved tooth alignment, both of which contribute to less mechanical stress on the periodontal tissues. Both the GTR and GTRO groups also showed significant reductions in PD and CAL, indicating the efficacy of GTR in promoting periodontal regeneration. However, the GTRO group exhibited significantly greater reductions in depth of bone defect, suggesting that orthodontic treatment plays an important role in facilitating bone repair and remodeling. This finding is consistent with previous research indicating that orthodontic therapy can enhance bone remodeling by improving tooth alignment and reducing occlusal trauma (Nangia et al., 2024). The improved alignment and function of the teeth may reduce the forces that hinder bone healing, thereby facilitating improved bone healing. The GTRO group’s greater reduction in depth of bone defect highlights the potential for orthodontic therapy to support bone defect repair in periodontal therapy. Orthodontic treatment may help reduce the irregular forces applied to the periodontium, providing a more stable environment for bone healing and tissue regeneration (Albardawel et al., 2024). This synergistic effect of GTRO may contribute to more effective treatment of periodontal bone defects.

A key finding of this study is the significantly greater reduction in inflammatory markers, particularly NLRP3, IL-1β, and IL-18, in the GTRO group compared to the GTR group. The NLRP3 inflammasome is a critical regulator of macrophage-mediated inflammatory responses (Kelley et al., 2019; Zhang et al., 2020). Its activation is influenced by pathogen signals and genetic polymorphisms that enhance NLRP3 transcription, which significantly contributes to periodontitis risk (Isaza-Guzman et al., 2016; de Alencar et al., 2020). Notably, specific NLRP3 polymorphisms are associated with heightened susceptibility to periodontitis, with males being at a greater risk than females (Honma and Honma, 1988; Isaza-Guzman et al., 2016). NLRP3 and AIM2 inflammasomes in gingival fibroblasts are differentially regulated by biofilms (Bostanci et al., 2011). *F. nucleatum* upregulates NLRP3 and AIM2 with increased IL-1β, while *Porphyromonas gingivalis* downregulates NLRP3 without affecting AIM2 (Aral et al., 2022). *In vitro* studies showed that low-level subgingival biofilms increased GFs' expression of NLRP3, caspase-1, ASC, AIM2, IL-1β, and IL-18, while high-level biofilms suppressed these genes (Bostanci et al., 2011). NLRP3 inflammasome activation plays a central role in the inflammatory process of periodontitis, leading to the release of pro-inflammatory cytokines such as IL-1β and IL-18, which are involved in tissue destruction. The observed reduction in these inflammatory markers in the GTRO group indicates that GTRO may exert a more pronounced anti-inflammatory effect than GTR alone.

Orthodontic forces may directly influence NLRP3 inflammasome activity by altering the periodontal microenvironment. Compressive force has been shown to activate the NLRP3 inflammasome in macrophages during orthodontic tooth movement via the cGAS/P2X7R axis (Han et al., 2022), whereas cyclic tensile stretch may inhibit NLRP3-dependent IL-1β secretion (Maruyama et al., 2019), suggesting that the effect on NLRP3 signaling depends on the type and magnitude of force. Additionally, hypoxia-related oxidative stress can activate the ROS/TXNIP/NLRP3 pathway in periodontal ligament cells (Zhu et al., 2022), while cyclic stretch-stimulated periodontal ligament cells may suppress macrophage NLRP3 activation through exosome-mediated signaling (Wang et al., 2019). Therefore, adjunctive orthodontic treatment may contribute to the reduced levels of NLRP3, IL-1β, and IL-18 observed in the GTRO group by optimizing the local biomechanical and inflammatory microenvironment. However, this hypothesis needs to be validated by further mechanistic investigation.

Importantly, growing evidence suggests that NLRP3 inflammasome-related cytokines, including IL-1β and IL-18, are involved not only in local periodontal inflammation but also in systemic inflammatory status. Systemic conditions associated with cytokine dysregulation, such as diabetes and other chronic inflammatory diseases, may affect periodontitis progression, treatment response, and prognosis (Hajishengallis, 2015; Graves et al., 2020). A recent review further suggested that dysregulated interleukins in systemic disorders, particularly type 1 diabetes, may influence periodontal inflammation and treatment outcomes, underscoring the importance of cytokine assessment beyond local periodontal tissues (D'Agostino et al., 2024). In this study, only local cytokines in GCF were evaluated. Although GCF reflects site-specific inflammation, it may not fully capture the systemic inflammatory status relevant to periodontal healing. Future studies should include concurrent assessment of systemic biomarkers to better clarify the interaction between local and systemic immune responses during GTRO.

While the study provides valuable insights, it has several limitations. First, the retrospective design limits the ability to draw causal inferences. Although matching was performed to control for confounding factors, residual confounding cannot be excluded because some potentially important variables, including smoking history, systemic diseases, and oral hygiene compliance, were not included in the propensity score matching model. In addition, this was a single-center study with a relatively small sample size, which may limit the generalizability of the findings. Prospective, randomized controlled trials are needed to further minimize confounding bias and confirm the long-term benefits and mechanistic pathways of GTRO. Second, inflammatory marker concentrations in GCF were not normalized to total protein. Although all samples were collected and processed under standardized conditions, the effects of variation in GCF volume cannot be fully excluded, and the findings should be interpreted with caution. Third, while the study focused on inflammatory markers and clinical outcomes, further research into other mediators of tissue regeneration, such as growth factors, would provide a more comprehensive understanding of the underlying biological mechanisms. Moreover, no formal correction for multiple comparisons was applied, which may have increased the risk of false-positive findings. In addition, the 6-month follow-up period may be insufficient to fully evaluate true periodontal regeneration and long-term stability. Future studies with longer follow-up durations, at least 12–24 months, are needed to assess the durability of the clinical improvements and the prevention of relapse in periodontitis patients.

## Conclusion

In conclusion, GTRO was associated with better clinical outcomes than GTR alone in periodontitis. GTRO improved recovery, gingival health, and reduced inflammatory markers such as NLRP3, IL-1β, and IL-18. Additionally, GTRO showed greater improvement in depth of bone defect and clinical attachment loss within the 6-month follow-up period, suggesting potential benefits for functional and aesthetic outcomes.

## Data Availability

The raw data supporting the conclusions of this article will be made available by the authors, without undue reservation.
